# New insights into the roles of the *FOXO3* and *P27Kip1* genes in signaling pathways

**DOI:** 10.1080/03009734.2019.1623351

**Published:** 2019-07-11

**Authors:** Sabah Mayahi, Masood Golalipour, Ahad Yamchi, Gagan Deep Jhingan, Majid Shahbazi

**Affiliations:** a Department of Molecular Medicine, School of Advanced Technologies in Medicine, Golestan University of Medical Sciences, Gorgan, Iran;; b Department of Medical Mycology and Parasitology, School of Medicine, Mazandaran University of Medical Sciences, Sari, Iran;; c Medical Cellular and Molecular Research Center, Golestan University of Medical Sciences, Gorgan, Iran;; d Department of Biotechnology, Gorgan University of Agricultural Sciences and Natural Resources, Gorgan, Iran;; e VProteomics, Ground Floor Green Park Main, New Delhi, India;; f AryaTinaGene Biopharmaceutical Company, Gorgan, Iran

**Keywords:** Breast cancer, cell signaling, FOXO3a, gene therapy, P27Kip1, proteomics

## Abstract

**Background:** The forkhead box O3 (FOXO3) and p27Kip1 are two important genes in breast cancer progression. In the present study we analyzed the effect of simultaneous FOXO3 silencing and p27Kip1 activation on breast cancer cell survival and the potential targets of these changes in cancer molecular pathways.

**Materials and methods:** The present study involved the cloning of *FOXO3a* shRNA and *p27Kip1* genes under the control of the bidirectional survivin promoter to down- and up-regulate *FOXO3* and *p27Kip1* genes, respectively. After transfection of the recombinant expression vector into the breast cancer cell line, the inhibition of cell growth was assessed by MTS and flow cytometry assays. Following the extraction of total mRNA and protein, the expression of target genes was evaluated by qPCR and Western blotting in both treated and untreated cell lines. Then, the downstream protein responses were examined by 2 D electrophoresis. The differentially expressed proteins were also identified by mass spectrometry.

**Results:** Rates of cell proliferation were significantly inhibited in the transfected cell line 72 h post-transfection. Proteomic profiling of the cell line resulted in the identification of seven novel protein markers in breast cancer responsive to these changes in expression of *FOXO3* and *p27Kip1*. The changes in expression of these markers suggested that certain signaling pathways contribute to the development of breast cancer.

**Conclusion:** Simultaneous silencing of *FOXO3* and activation of *p27Kip1* in MDA-MB-231 cells caused alterations in the expression level of several genes involved in apoptosis, cell proliferation, cell cycle control, tissue invasion, drug resistance, and metastasis. It seems that the identified genes might serve as useful biomarkers for breast cancer.

## Introduction

Breast cancer is an important global health concern and the second leading cause of cancer-related mortality among women (1). Omics studies have revealed several pathways and genes related to breast cancer (2), such as *p27Kip1* and forkhead box O3 (*FOXO3*). The p27Kip1 protein interacts with cyclin E and cyclin-dependent kinase 2 (*CDK2*). In addition, the *p27Kip1* gene arrests the progression of the cell cycle and leads to apoptosis, tumor suppression, and cell adhesion (3). Poor prognosis in patients with colorectal, gastric, pulmonary, and breast cancers is associated with a low expression level of the *p27Kip1* gene (4).

The FOXO proteins, a subgroup of forkhead transcription factors, are involved in apoptosis, differentiation, stress response, cell cycle control, and cell metabolism (5). Overall, the *FOXO3* gene is involved in many signaling pathways and makes major contributions to several processes, including cancer development (6). Therefore, the identification of *FOXO3* inhibitors presents a promising strategy for future anticancer drugs (7). Proteomic analysis is generally applied in breast cancer serving two ends, namely the identification of novel molecular markers for breast tumor profiling and determination of the intracellular signaling pathways contributing to the growth of breast cancer cells (8). To our knowledge, there is no information on the effect of simultaneously decreasing *FOXO3* gene and increasing *p27Kip1* gene expression, respectively, to inhibit breast cancer. With this background in mind, the present study aimed to analyze the effect of the *FOXO3* and *p27Kip1* genes on the control of the breast cancer MDA-MB-231 cell line, using a bidirectional survivin promoter. Moreover, the changes in the protein profile of the transfected cell line were investigated in order to find cancer molecular markers.

## Materials and methods

### Cell line, plasmid constructs, and transfection

The MDA-MB-231 cell line adopted in this study was supplied by the Pasteur Institute (Tehran, Iran). Cells were cultured in a high-glucose Dulbecco’s modified Eagle’s medium (ThermoFisher scientific, Waltham, MA, USA), supplemented with penicillin-streptomycin (100 units/mL) and fetal bovine serum (10% FBS; Gibco BI102-100) in a damp atmosphere (5% CO_2_) at 37 °C. The cells were transfected with a plasmid construct designed in a bidirectional promoter (dual targeting of *FOXO3a* shRNA and simultaneous induction of *p27Kip1* gene).

**Figure 1.**
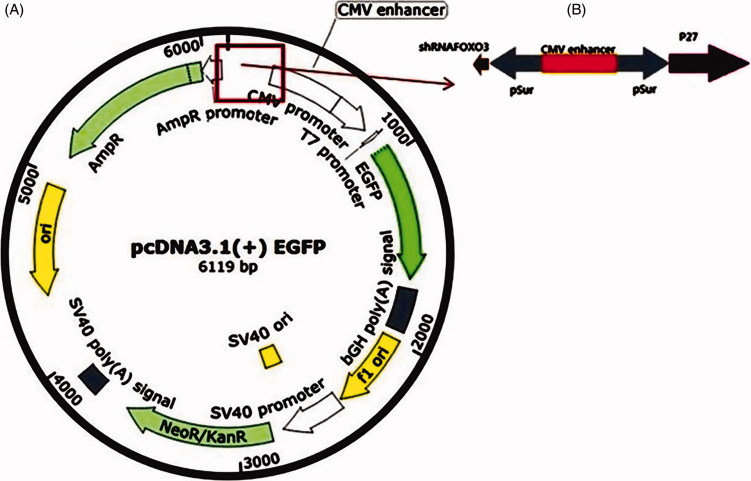
(A) Map of pcDNA3.1(+) EGFP; (B) pSur (*shFOXO3-p27Kip1*) vector with a survivin promoter.

In addition, an empty plasmid of pcDNA3.1^+^ expression vector with EGFP was used as the control (Figure 1). The vectors were purchased from BioMatic, Toronto, Ontario, Canada. To verify the identity of the inserted sequences, all plasmids were examined through direct sequence analysis. Twenty-four hours before transfecting DNAs into the cells, some of the cells were applied in the growth medium without antibiotics. Transfection was performed as recommended by the manufacturer using Lipofectamine 2000 (Invitrogen, Waltham, MA, USA).

### Cell viability assay

The viability of the cells (2–5 × 10^3^/well) was tested in a 96-well plate in a final volume of 200 μL/well, incubated in a CO_2_ incubator (5% CO_2_) at 37 °C overnight. Then, 20 μL/well of MTS reagent (Promega, Fitchburg, WI, USA) were added to each well and incubated for 4 h at 37 °C. The plate was shaken briefly, and then the absorbance of the treated and untreated cells was measured at 490 nm 24 h, 48 h, and 72 h after transfection, using a plate reader (BioTek, Winooski, VT, USA).

### Quantitative real-time polymerase chain reaction assay

The extraction of total RNA was accomplished using TRIzol solution (Invitrogen). Total RNA (2 µg) was used for complementary DNA synthesis as recommended by the manufacturer (Thermo Scientific, Vilnius, Lithuania). Quantitative real-time reverse transcriptase polymerase chain reaction (qPCR) assay was performed using ABI 7300 RT-PCR systems (Applied Biosystems, Foster City, CA, USA) to determine the relative expression of the *p27Kip1* and *FOXO3a* genes (Table 1). The threshold cycle (Ct) value was estimated, and the REST 2009 software was used to assess the amplification plots. In addition, the comparative Ct (2^−ΔΔCt^) method was applied to analyze the data (8).

### Apoptosis assay

To identify apoptotic cells, the FITC Annexin V Detection Kit with propidium iodide (PI) (BioLegend, San Diego, CA, USA) was exclusively developed. After seeding the cells (2 × 10^5^ cells/mL) in tissue culture plates, they were incubated overnight, and then treated after 24 h, 48 h, and 72 h. In the next stage, the cells were washed with a cell-staining buffer, and then re-suspended in the annexin V binding buffer at a concentration of 0.25–1.0 × 10^7^ cells/mL. Subsequently, the PI solution (10 μL) and FITC annexin V (5 μL) were added. The cells were vortexed and incubated at 25 °C for 15 min in darkness. Then, the annexin V binding buffer (400 μL) was added to the tubes. Finally, a BD Accuri™ C6 cytometer (Seattle, WA, USA) was used to perform flow cytometry.

### Protein extraction and immunoblotting

A protein lysis buffer (100 mM Tris-HCl, 150 mM NaCl, 5% SDS, 1 mM dithiothreitol, 5 mM EDTA, pH of 7.4, 5% sodium deoxycholate, and 10% glycerol) was used for lysing the harvested cells collected via trypsinization. A protease inhibitor cocktail (Sigma-Aldrich, St. Louis, MO,USA) mixture was added to 1 mM of PMSF. The total protein in the resultant supernatant was used for Western blotting analysis. After separating the total protein using polyacrylamide gel electrophoresis, it was transferred to a nitrocellulose membrane, which was blocked with bovine serum albumin (5% w/v bovine serum albumin; Sigma-Aldrich) in TBST. Furthermore, the primary antibody was used against *FOXO3a* and *p27Kip1* antibodies (Santa Cruz, Santa Cruz, CA, USA). Visualization of the bands was accomplished by means of the Enhanced Chemiluminescence Detection Kit (Parstous, Tehran, Tehran, Iran) following the manufacturer’s protocol. In addition, the National Institutes of Health ImageJ program (Bethesda, MD, USA) was used to determine the band density.

### Two-dimensional gel electrophoresis

Cell pellets were resuspended by adding an adequate amount of 2 D lysis buffer, containing 2 M thiourea, 1% dithiothreitol, 4% CHAPS, 7 M urea, 0.001% bromophenol blue, and 0.5% immobilized pH gradient (IPG) buffer (pH of 3–10; GE Healthcare, USA), followed by extracting the total protein. In addition, a 2 D Quant Kit (GE Healthcare) was used to measure the protein level with bovine serum albumin as the standard. To this end, after rehydrating the IPG strips (7 cm 3–10 NL, Immobilone Dry Strip; GE Healthcare) at a pH gradient of 3–10, 300 µg of each protein sample was loaded on the IPG strip. The 2 D electrophoresis was carried out based on our previously published method (9). For staining the gels, a colloidal Coomassie Brilliant Blue dye was used as described by Candiano et al. (10). A scanner (GE Healthcare Life Sciences, Chicago, IL, USA) was also employed to scan the stained gels. The analysis of the protein spots in the gel images was performed by the ImageMaster 2 D Platinum 6.0 (GE Healthcare Life Sciences). Finally, the intensities of the treated and untreated gels were compared, and their ratios were determined.

### Protein identification via liquid chromatography-mass spectrometry

After cutting the protein spots into 1-mm^3^ pieces, they were removed from the stained gel manually and added to tubes. The pieces were destained with an equal solution of ammonium bicarbonate and acetonitrile. Then, they were digested with trypsin (Promega, USA) at 37 °C for a minimum of 16 h. Trifluoroacetic acid (TFA) 10% was also added to prevent digestion. Extraction of the gel pieces was accomplished using an equal solution of 0.1% TFA and acetonitrile (50 μL).

Supernatants from the pooled samples were dried, using Speed-Vac centrifugation (Thermo Scientific, Waltham, MA, USA). Then, 2% acetonitrile (10 μL) and 0.1% TFA were used for dissolving the samples. The Q-TRAP nLC-MS/MS system (Applied Biosystems) was used for all analyses, as described (11). Mass data were collected and analyzed using the Analyst software (Applied Biosystems). For finding the SwissProt-Trembl subdatabase, the MS/MS lists were searched, and Mascot 3 (version 2.2., Matrix Science, Boston, MA, USA) was used to blast the data lists against databases.

### Data analysis

Data were analyzed in Prism, version 6.0 (GraphPad Software Inc, San Diego, CA, USA) using two-way ANOVA and Student’s *t* test. The data were also presented as mean ± standard error of mean of at least three independent analyses. A *p* values less than 0.05 was considered statistically significant.

## Results

### Combinatorial effects of induced p27Kip1 and shFOXO3a on cell viability

**Figure 2.**
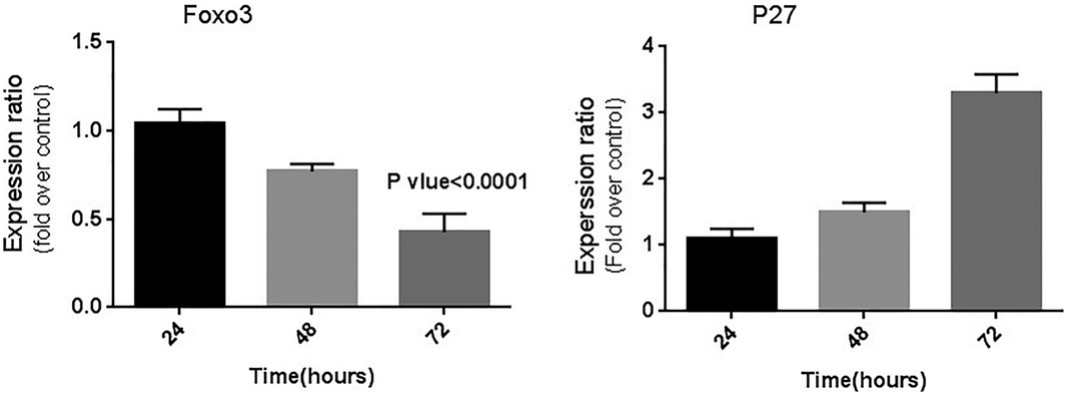
Relative expression of *FOXO3* and *p27Kip1* genes in the MDA-MB-231 cell line after transfection was significantly down-regulated and up-regulated, respectively, 72 h after transfection. Expression of the genes was analyzed by the REST program (*P* < 0.05).

The *p27Kip1* mRNA expression level was significantly higher, and *FOXO3a* mRNA expression significantly lower, in treated cells 72 h post-transfection in comparison with that found in controls (*P* < 0.05). Analysis of the qPCR results showed a 0.41-fold change in *FOXO3a* gene expression (down-regulation) and more than a three-fold change in *p27Kip1* gene expression (up-regulation) 72 h after transfection (Figure 2).

**Figure 3.**
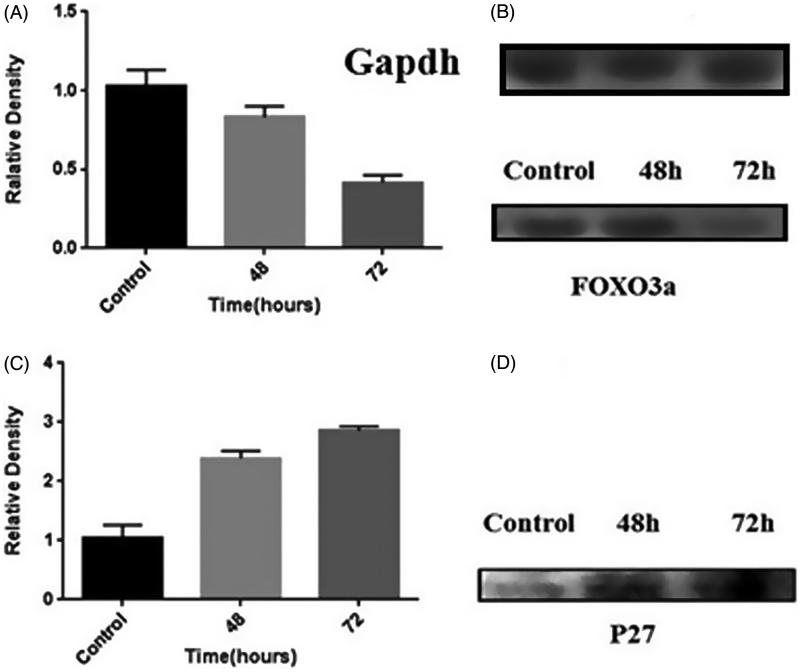
Relative band densitometry of Western blotting data, showing: (A) the incidence of the highest down-regulation of *FOXO3a* gene 72 h after transfection; (B) reduction of *FOXO3a* gene expression in the cells treated with the construct after 72 h, compared to the control; (C) the highest up-regulation of *P27Kip1* gene in MDA-MB-231 cells occurring 72 h post-transfection; and (D) an increase in *P27Kip1* expression in the cells treated with the construct 72 h after transfection, compared to the control. Implementation of quantification in three repeats (GAPDH was used as the control).

*FOXO3* expression decreased (60%), while *p27Kip1* expression increased 72 h after transfection, compared to the control expression (Figure 3).

**Figure 4.**
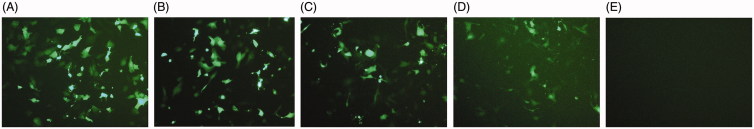
Microscopic morphology of MDA-MB-231 cells under a fluorescent microscope after transfection for 24 h, 48 h, and 72 h with: (A) an empty vector; (B) a vector containing the genes after 24 h, (C) 48 h, (D) 72 h; and (E) negative signal of control cells without transfection.

The expression of construct in MDA-MB-231 significantly reduced the growth of the cell line (*P* < 0.05) (Figure 4).

**Figure 5.**
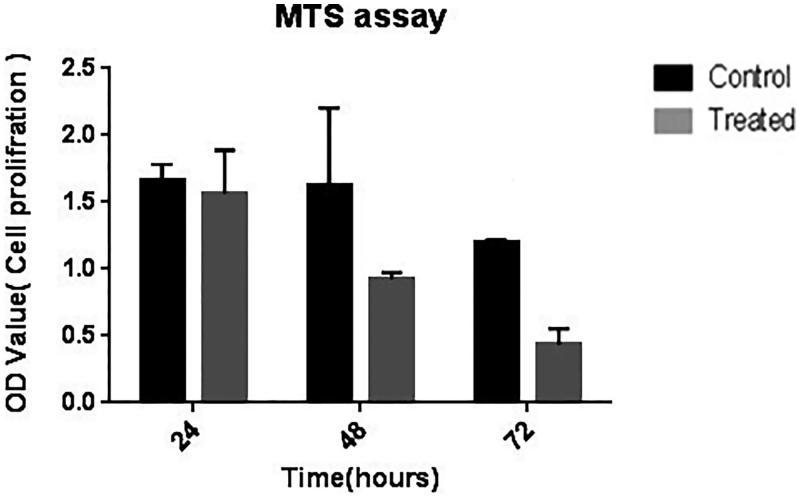
Inhibition of MDA-MB-231 cell proliferation after 24 h, 48 h, and 72 h of treatment in wells (in triplicate) using MTS assay. Data are expressed as the mean cell viability percentage versus that of the control.

The effect of the construct on the rate of cell proliferation was not statistically significant 24 h post-transfection. However, it was significantly inhibited at 72 h after treatment (*P* < 0.05; Figure 5).

### Flow cytometry

**Figure 6.**
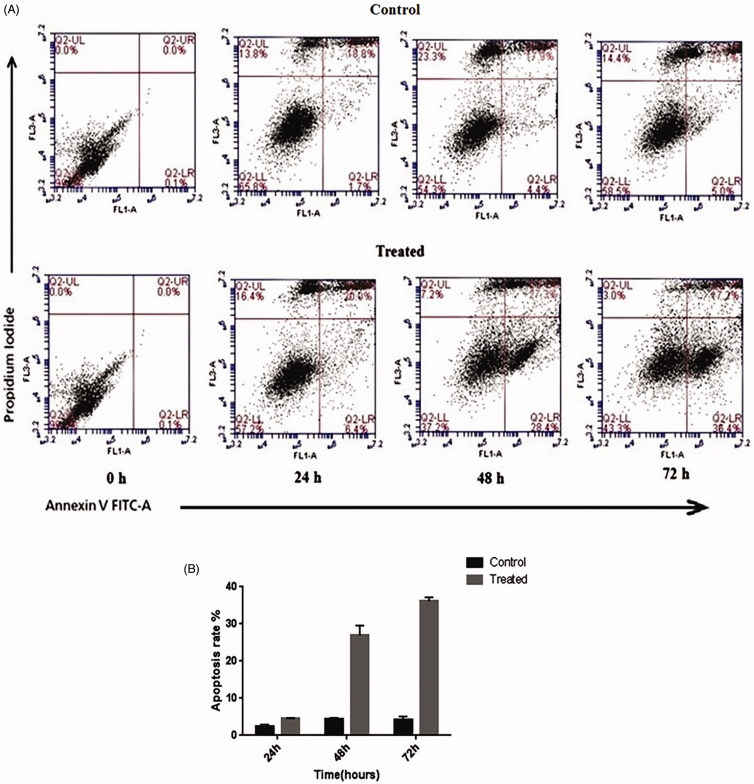
Apoptosis detection via flow cytometry. A: use of Annexin V-FITC staining in the treated and untreated MDA-MB-231 cells after 24 h, 48 h, and 72 h. The upper and lower right quadrants (Annexin V+/PI + and Annexin V+/PI−, respectively) indicate late and early apoptosis, respectively (10.4% of cells died by lipofectamine, as shown in Supplementary Figure 1, available online). B: Apoptosis in the treated and control MDA-MB-231 cells 24 h, 48 h, and 72 h post-transfection. Data were presented as mean ± SD (*P* < 0.05).

Apoptotic cells included both early and late apoptosis (Annexin V+/PI- and Annexin V+/PI+, respectively), while Annexin V and PI were negative for viable cells. In the controls, the majority of the cells (99.9%) were viable and non-apoptotic at baseline. However, early apoptotic cells showed a significant increase after treatment, compared to the untreated cells after 72 h (from 5.0% to 36.4%; Annexin V+/PI–; Figures 6(A, B)).

### Effect of overexpression p27Kip1 and FOXO3a shRNA on downstream proteins

**Figure 7.**
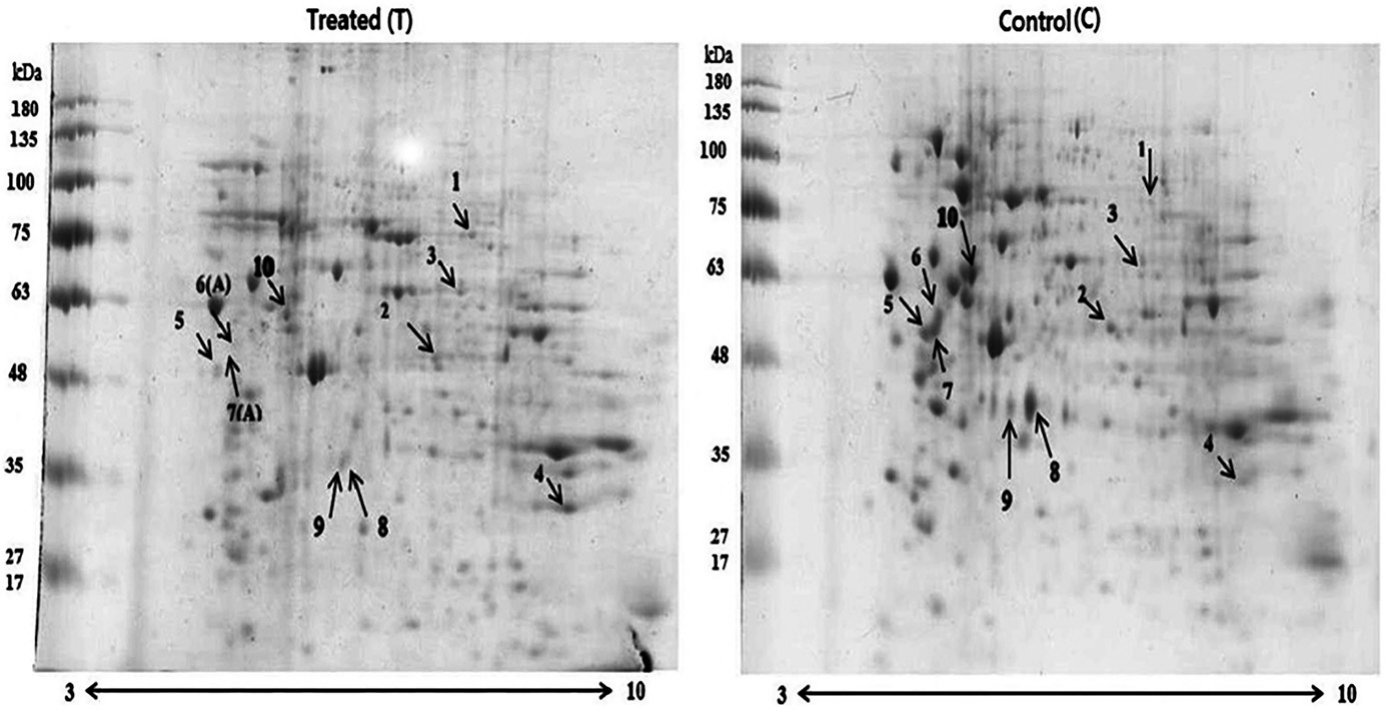
Two-dimensional gel electrophoresis of MDA-MB-231 cell line in treated and untreated cells, showing the increased expression of tubulin beta chain, vimentin (three isoforms), and β-actin (two isoforms) and decreased expression of succinate dehydrogenase complex, albumin, and NLK. Protein spots 6 and 7 were absent in the treated cells. The experiment was performed in two biological repeats. A = absence.

To identify the downstream responsive proteins associated with the changes in *p27Kip1* and *shFOXO3a* expression at 72 h after transfection, the treated and untreated samples were compared by 2 D gel electrophoresis. There was differential expression of 10 protein spots in the breast cancer cell line (Figure 7). Out of the 10 proteins, 3 and 7 proteins showed up-regulated and down-regulated expression, respectively (Figure 7). Table 2 presents the mass spectrometry (MS) information of the appeared spots. Succinate dehydrogenase (SDH; T1/C1), albumin (ALB; T3/C3), and serine/threonine-protein kinase NLK (NLK; T4/C4) were significantly up-regulated, while tubulin alpha (TUBA1C; T2/C2), tubulin beta chain (TUBB; T10/C10), β-actin (ACTB) with two isoforms (T8/C8 and T9/C9), and vimentin (VIM) with three isoforms (T5/C5, T6/C6, and T7/C7) were significantly down-regulated (Figure 7). The theoretical and experimental masses and the isoelectric points of these differentially expressed proteins were also nearly the same, verifying the identified MS results (Table 2). Gene ontology (GO) analysis of 10 differentially expressed proteins consisted of protein–protein interactions using the GeneMANIA (https://genemania.org) and KEGG (https://www.genome.jp/kegg/kegg2.html) databases (Table 3). Based on the result of two-way ANOVA, after the transfection of genes in MDA-MB-231 cells for 72 h, *FOXO3* gene expression showed the greatest reduction (*P* < 0.05), while *P27Kip1* gene showed the highest increase, compared to the control (Figure 7). The 2 D gel electrophoresis of the plasmid construct and empty vector-transfected MDA-MB-231 cells indicated changes in the expression level. Out of the collected 25 spots, 10 spots (indicated by arrows, Figure 7) that had the highest scores were selected and confirmed to be involved in breast cancer.

**Table 1.** Primers used in this study.

Primer	Sequence (5’→3’)
FOXO3a (R)	GACTATGCAGTGACAGGTTGTG
FOXO3a (F)	TCTACGAGTGGATGGTGCGTT
P27 (R)	TCCATTCCATGAAGTCAGCG
P27 (F)	GAGCAGACGCCCAAGAAGC
GAPDH (R)	AGGCAGGGATGATGTGGAGAG
GAPDH (F)	ATCGTGGAACTCAGTACCACA

**Table 2.** Protein spots collected from two-dimensional gels in MDA-MB-231 cell line transfected with the plasmid construct in comparison with the control (empty vector) after 72 h.

Spot ID	Accession	Name of protein	Gene	Theoretical MW (kDa)/PI	Experimental MW (kDa)/PI	Ratio	Peptide	Score
1	P31040	Succinate dehydrogenase	SDHA	72.6/7.39	72.2/7.8	2.1 (up-regulated)	VGSVLQEGGK	2.65
2	Q9BQE3	α-Tubulin	TUBA1C	51/6.0	49.9/7.1	0.2 (down- regulated)	AVFVDLEPTVIDEVR; EIIDLVLDR; EDAANNYAR; DVNAAIATIK; TIGGGDDSFNTFFSETGAGK; LSVDYGKK; LSVDYGK	12.49
3	P02768	Albumin	ALB	69.3/6.28	65.1/6.5	1.2 (up-regulated)	KVPQVSTPTLVEVSR; AEFAEVSK; LVTDLTK	2.49
4	Q9UBE8	Serine/threonine-protein kinase NLK	NLK	8.13/58.28	34..6/8.7	4.5 (up-regulated)	TQEVVTQYYRAPEILMGSRHYSNAIDIWSVGIFAELLGR; PNVFQNLVSCK; ICDFGLARVEELDESR	2.6
5	P08670	Vimentin	VIM	53.65/5.12	49.8/5.5	0.3 (down-regulated)	QDVDNASLAR; QVQSLTEVDALK; VELQELNDR; QQYESVAAK; EEAENTLQSFR; ETNLDSLPLVDTHSK; EYQDLLNVK; LQEEMLQR;	19.82
6	P08670	Vimentin	VIM	53.65/5.12	52.5/5.7	a/p (down-regulated)	EEENFAVEAANYQDTIGR; ALDIEIATYR; EKLQEELQR; QVQSLTEVDALK; QDVDNASLAR; VESLQEEIAFLK; VELQELNDR; EEAENTLQSFR; ETNLDSLPLVDTHSK; QQYESVAAK; LQEEMLQR; LLEGEESR; DNLAEDIR; QVDQLTNDK	35.71
7	P08670	Vimentin	VIM	53.65/5.12	50.4/5.6	a/p (down-regulated)	VELQELNDR; FADLSEAANR; QYESVAAKLGDLYEEEMR; EAENTLQSFR; ALDIEIATYR; LQEEMLQR; QESTEYR; ETNLDSLPLVDTHSK; QVQSLTEVDALK; LGDLYEEER; DNLAEDIR; LLEGEESR; LQDEIQNKEEAR; FANYIDK	27.58
8	P60709	β-Actin	ACTB	41.7/5.48	40.5/6.5	0.2 (down-regulated)	QEYDESGPSIVHR;GYSFTTTAER; DLTDYLMK; APEEHPVLLTEAPLNPK; DSYVGDEAQSKR; DSYVGDEAQSK; EITALAPSTK; DLTDYLK; DLYANTVLSGGTTYPGIADR	17.02
9	P60709	β-Actin	ACTB	41.7/5.48	40.89/6.7	0.1 (down-regulated)	QEYDESGPSIVHR;GYSFTTTAER; DLTDYLMK; APEEHPVLLTEAPLNPK; DSYVGDEAQSKR; DSYVGDEAQSK; EITALAPSTK; DLTDYLK; DLYANTVLSGGTTYPGIADR	10.72
10	P07437	Tubulin beta chain	TUBB	51.2/5	49.6/5.8	0.3 (down-regulated)	INTFSVVPSPK; TAVDIPPR; ISVYYNEATGGK; NAADPR; LAVNVPFPR	46.38

a/p: absence/presence; MW: molecular weight.

**Table 3.** Protein–protein interactions of all proteins for collecting the data.

	Gene						
	Vim	ACTB	TUBA1C	TUBB	SDHA	NLK	ALB
Expand							
Physical interaction	SERPINH, MAGOH, EIFA3, SPRM4, KRT18, UPP1, ITGB4, BASP1, PPL, URGDP, XPNPEP3	CFL1, CFL2, PFN1, CCT8, TCP1, CCT3, CCT4, RUVBL1, MYH11	TUBA4B, TUBB6, TUBB, TUBA4A, TUBB4A, TUBB4B, TUBB3, TUBB2A, TUBB6, TUBB2B, GAPDH,	THBS1, CLIP1, TCP11L1, TUBB2A, YBX1, TUBA1A, TUBA1B, TUBA1C, TUBA1A, TUBA4B, PNKD, TNPO2, DNAJA4	SLC2A14, CPNE1, RAB14, LRRC8A, HIGD1A, SDHAF2, SDHAF2, SDHC, SDHB, UBC	MYB, RANGAP1, ZHX3, MAP3K7, TAB1, HIPK2, LEF1, TP53, TCF7L2	F2, HP, ITIH1, AMBP, AHSG, APOA2, RBP4, FCGRT
Co-expression	SERPINH, TTN	CFL1, PFN1	TUBB6, TUBB, TUBA4A, TUBB4B, TUBB3, TUBB2A, TUBB6, TUBB2B, GAPDH, TUBA1A, TUBA1B, TBCB, PFDN1, STMN1	TUBAB, TUBB2A, TUBG2, YBX1, TUBA1B, TUBA1C, TUBA4A, TUBB3, TUBB4B, TUBG1	RAB14, HIGD1A, SDHC, SDHB, NDUFV1, UBC, ALAD, UQCRC1	—	F2, HP, ITH1, GC, AMBP, AFM, AHSG, AFP, APO2, RBP4, SLCO1B1, SLCO1B3
Co-localization	SERPINHI	—	STMN1, TBCB, TUBA1B, TUBA1A, TUBB2A	—	NDUFV1, UQCRC1, NDUFS1	RANGAP1, MAP3K7, TAB1	F2, HP, ITH1, GC, AMBP, AHSG, APO2, RBP4, SLCO1B1, SLCO1B3
Gene interaction	—	—	—	—	—	TP53	—
Role	Proliferation, tissue invasion, and metastasis	Cell mobility, endocytosis, cell adhesion, division, drug resistance, and metastasis	Cellular mobility	Cellular mobility	Tumor suppressor	Inhibit cellular proliferation, induce apoptosis	Stabilizing DNA, regulation calcium, antioxidant properties

## Discussion

**Figure 8.**
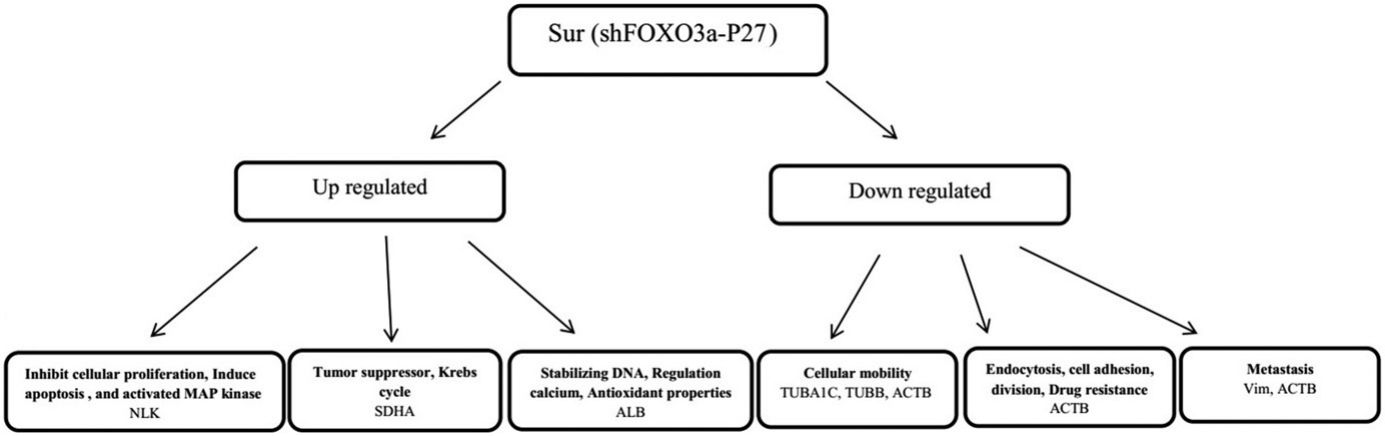
Effect of simultaneous *FOXO3a* suppression and *P27Kip1* induction on protein expression in treated cells.

There are studies regarding the independent effect of *P27Kip1* and *FOXO3a* genes on the inhibition of breast cancer; however, they have reported a poor prognosis (12,13). The present study involved the design of a bidirectional expression vector suppressing *FOXO3a* and inducing *p27Kip1* expression. Our results showed that the synergistic effects of *p27Kip1* and *shFOXO3a* induced 36.4% apoptosis in cells. However, the overexpression of *p27Kip1* alone has been reported to induce 12.73% apoptosis (12). Recently, proteomic methods have been used as a strategy for the detection of protein biomarkers (14). Expression of seven proteins changed after *FOXO3a* suppression and *p27Kip1* induction (Figure 8). These proteins are all involved in apoptosis, metastasis, drug resistance, proliferation, and cell cycle control.

Three up-regulated proteins included SDHA, albumin (with cell source), and NLK, while four down-regulated proteins included TUBA1C, TUBB, ACTB with two isoforms, and VIM with three isoforms. The SDH is identified as a mitochondrial enzyme, involved in the Krebs cycle and electron transfer chain, and contributes to tumor suppression. This enzyme has four subunits, namely SDHA, SDHB, SDHC, and SDHD (15). Research over the past decades has shown an inverse relationship between albumin level and severity/risk of the progression of different cancers, including pulmonary, gastric, ovarian, and breast cancers. There are various mechanisms for the anticancer effects of albumin, such as DNA stabilization and duplication, calcium preservation and regulation, and antioxidant properties. In a study performed by Xiao-Jing Du et al., the growth of human cancer cells, such as breast cancer cells, was inhibited by high concentrations of albumin (16). The main cause of the reduction of the albumin level, especially in cancer patients, is the inhibition of albumin-regulating gene by tumor necrosis factor which results in mRNA expression reduction of approximately 90% in the liver (17). NLK, which belongs to the mitogen-activated protein kinase family, is a vital regulator of various cancer types. In a study conducted by Lv et al., NLK inhibited non-small-cell lung cancer through the Wnt signaling pathway (18). In addition, Huang et al. showed that NLK inhibits cellular proliferation in breast cancer and induces apoptosis. Similar studies confirm that NLK is a tumor suppressor gene in ovarian cancer (19). The theoretical molecular weight of NLK is about 58 kDa, while its experimental molecular weight is about 35 kDa, which can be attributed to the degradation of this protein. Actin and its proteins have a major functional and structural role in cell motility, endocytosis, cell adhesion and division, and maintenance of cell morphology. The expression of ACTB increases in a metastatic state (20). Accordingly, Chunmei Guo et al. reported that ACTB increased in many cancers of the liver, kidneys, colon, stomach, pancreas, esophagus, lung, breast, prostate, and ovaries (21). These tumors are associated with increased drug resistance and metastasis, especially in MDA-MB-231 cells (21).

Tubulins consist of microtubules, with two alpha and beta subunits (approximate weight of 50 kDa). They play a large role in cellular activities, such as cellular mobility. Dysfunction of the microtubule network causes disturbance in apoptosis and differentiation (20). In addition, VIM is one of the most filamentous proteins, expressed in various types of cancers, such as prostate, gastrointestinal, central nervous system, pulmonary, and breast cancers. In a study carried out by Satelli, it was shown that the increased invasive/migratory potential of cancer cells is related to VIM overexpression (22). The VIM is generally considered as an indicator of poor prognosis (22).

In conclusion, the simultaneous silencing of *FOXO3* and activation of *p27Kip1* in MDA-MB-231 cells caused alterations in the expression level of several genes which are involved in cell apoptosis, cell proliferation, cell cycle control, tissue invasion, drug resistance, and metastasis. Therefore, it seems that these genes may serve as useful biomarkers for breast cancer.

## Supplementary Material

Supplemental Material
